# Significance of macrophage infiltration in the prognosis of lung adenocarcinoma patients evaluated by scRNA and bulkRNA analysis

**DOI:** 10.3389/fimmu.2022.1028440

**Published:** 2022-10-12

**Authors:** Huaiyang Zhu, Chunning Zheng, Hongtao Liu, Fanhua Kong, Shuai Kong, Feng Chen, Yuan Tian

**Affiliations:** ^1^ Department of Thoracic Surgery, Shandong Second Provincial General Hospital, Jinan, China; ^2^ Department of Gastrointestinal Surgery, Shandong Provincial Hospital affiliated to Shandong First Medical University, Jinan, China; ^3^ Department of Pathology, The First Affiliated Hospital of Shandong First Medical University and Shandong Provincial Qianfoshan Hospital, Shandong Medicine and Health Key Laboratory of Clinical Pathology, Shandong Lung Cancer Institute, Shandong Institute of Nephrology, Jinan, China; ^4^ Department of Thoracic Surgery, The Affiliated Taian City Centeral Hospital of Qingdao University, Taian, China; ^5^ Department of Thoracic Surgery, Shandong Cancer Hospital and Institute, Shandong First Medical University and Shandong Academy of Medical Sciences, Jinan, China; ^6^ Department of Otolaryngology-Head and Neck Surgery, Shandong Provincial ENT Hospital, Shandong University, Jinan, China; ^7^ Radiotherapy Department, Shandong Second Provincial General Hospital, Shandong University, Jinan, China

**Keywords:** macrophages, prognosis, lung adenocarcinoma, ScRNA, bulkRNA, infilitration, marker gene, WGCNA (weighted gene co- expression network analyses)

## Abstract

**Purpose:**

To investigate the significance of macrophage infiltration to the prognosis of lung adenocarcinoma.

**Methods:**

R language bioinformatics analysis technology, was used to obtain macrophage infiltration-related module genes through WGCNA (Weighted Gene Co-Expression Network Analysis). Marker genes of macrophage subtypes were identified using single-cell sequencing of lung adenocarcinoma tissue. Risk score models were constructed and validated using external data cohorts and clinical samples.

**Results:**

Analysis of cohorts TCGA-LUAD, GSE11969, GSE31210, GSE50081, GSE72094 and GSE8894, revealed a negative correlation between macrophage infiltration and survival. Immunohistochemical analyses of clinical samples were consistent with these data. Based on cell-cluster-markers and TAMs-related-genes, TOP8 genes were obtained (C1QTNF6, CCNB1, FSCN1, HMMR, KPNA2, PRC1, RRM2, and TK1) with a significant association to prognosis. Risk score models including 9 factors (C1QTNF6, FSCN1, KPNA2, GLI2, TYMS, BIRC3, RBBP7, KRT8, GPR65) for prognosis were constructed. The efficacy, stability and generalizability of the risk score models were validated using multiple data cohorts (GSE19188, GSE26939, GSE31210, GSE50081, GSE42127, and GSE72094).

**Conclusions:**

Macrophage infiltration negatively correlates with prognosis in patients with lung adenocarcinoma. Based on cell-cluster-markers and TAMs-related-genes, both TOP8 genes (C1QTNF6, CCNB1, FSCN1, HMMR, KPNA2, PRC1, RRM2, TK1) and risk score models using C1QTNF6, FSCN1, KPNA2, GLI2, TYMS, BIRC3, RBBP7, KRT8, GPR65 could predict disease prognosis.

## 1 Introduction

Lung cancer remains the most common malignancy worldwide and a leading cause of cancer-related death, despite advances in screening and treatment (1, 2). Whether it was for the non-small cell lung cancer (NSCLC) or small cell lung cancer (SCLC) patients, immunotherapy was the most shining one among many treatment methods, which had changed the landscape of anti-tumor therapy and brought anti-tumor therapy into a new era (3–7). However, there were still many details in the screening of immunotherapy benefit populations and related predictors needed to be further elucidated (8–16). Specific macrophage phenotypes can act as indicators of lung cancer prognosis and the efficacy of immunotherapy (17–24). Sequencing technologies and R language based bioinformatics, formerly reported (25–27), can be used for studies in this area (28–30). Based on our previous studies (28–30), we performed bioinformatics analysis and clinical sample validation to identify specific macrophage signatures that can act as indicators of therapeutic efficacy.

## 2 Methods

### 2.1 Data analysis

#### 2.1.1 TCGA data

mRNA expression profiles, clinical information, copy number alterations and mutations of GDC TCGA Lung Adenocarcinoma (LUAD) samples were downloaded from https://xenabrowser.net/datapages/. Tumor samples were screened according to sample name. RNA-seq data for 513 tumor samples and 59 paracancerous samples were obtained.

#### 2.1.2 GEO data

Expression data and sample survival information for GSE11969, GSE19188, GSE26939, GSE31210, GSE42127, GSE50081, GSE72094 and GSE8894 were downloaded from the GEO database (https://www.ncbi.nlm.nih.gov/geo). Survival information of the samples were summarized as follows: (**Supplementary Table 1**_train_clin.tsv; **Supplementary Table 1**_ GSE11969_clin.txt; **Supplementary Table 1**_GSE19188_clin.txt; **Supplementary Table 1**_ GSE26939_clin.txt; **Supplementary Table 1**_GSE31210_clin.txt; **Supplementary Table 1**_ GSE42127_clin.txt; **Supplementary Table 1**_GSE50081_clin.txt; **Supplementary Table 1**_ GSE72094_clin.txt; **Supplementary Table 1**_ GSE8894_clin.txt). Single-cell sequencing data from GSE131907 were downloaded from the GEO database (https://www.ncbi.nlm.nih.gov/geo). A total of 42,995 cells and 29,634 genes were obtained.

### 2.2 Immune infiltration analysis

Immune infiltration for each sample was calculated using IOBR of the R package for the training set TCGA expression matrix and GEO data, respectively (method = ‘cibersort’).

### 2.3 Survival analysis

For survival assessments, R packages “survminer” and “survival” were analyzed and survival curves were constructed based on survival time and status. Differences in prognosis among the groups were assessed.

### 2.4 Screening of modules corresponding to macrophages using WGCNA

Hierarchical clustering analysis was performed on the TCGA expression matrix using the R package “hclust”, “method=average”. Phenotypic information was obtained using the infiltration ratio of macrophages. A correlation between different modules and macrophages was obtained.

### 2.5 Clustering analysis of samples

The R package “ConsesusClusterPlus” was used to perform consensus clustering analysis. After clustering on the TCGA and GEO data, the optimal number of categories were determined according to the change of area under the CDF curve. The k value of the cluster category ranged from 2 to 6.

### 2.6 Analysis of single-cell data

Single-cell data were filtered using the R package “seurat” to remove cells with ≥ 20% mitochondrial expression. Data were analyzed using the “seurat” normalization pipeline. To identify tumor-associated macrophage (TAM) populations, marker genes from published studies were used to identify corresponding clusters. TAM populations were selected for standardization analysis using “Seurat”.

### 2.7 Trajectory analysis of single-cell data

Trajectory analysis was performed on TAM subclasses using the R package “monocle” with default parameters. This resulted in differentiation trajectories and key genes determining these trajectories.

### 2.8 Gene set variation analysis

To investigate differences in the expression patterns of specific TAM isoforms in biological processes, GSVA enrichment analysis was performed using the R package “GSVA”. GSVA is a nonparametric, unsupervised method primarily used to assess alterations in signaling pathways and biological processes in samples.

### 2.9 Construction of risk scoring model

Univariate cox regression analysis was performed on “cell-cluster-markers” and “TAMs-related-genes”, and genes significantly associated with OS survival were screened at the p<0.05 level. According to the identified prognosis-related genes, the R package ‘glmnet’ was used to construct a prognosis model (or classifier model) with a 10-fold cross-validation fold using the cox method. Characteristic factors were then screened. Kaplan-Meier survival analysis and ROC curves were used to evaluate the predictive power of the prognostic model.

### 2.10 Clinical sample validation (sample collection and immunohistochemistry)

Lung Cancer samples were collected from the First Affiliated Hospital of Shandong First Medical University & Shandong Provincial Qianfoshan Hospital from June 2012 to February 2020. Written informed consent was provided by all participants. Tumor tissues were surgically resected, formalin fixed and paraffin embedded (FFPE) for histological evaluation. HE-stained and immunohistochemical (IHC) slides were examined by two independent and experienced pathologists according to the WHO criteria.

Samples were IHC stained with mouse anti-human CD68 monoclonal antibodies (MAB-0863, clone MX075) and mouse anti-human CD163 monoclonal antibodies (MAB-0869, clone MX081). CD68 was used as a general surface marker for macrophages, whilst CD163 was used as a marker for M2 macrophages (31). Double-labeled immunohistochemical staining was performed using alkaline phosphatase and horseradish peroxidase conjugated secondary antibodies. Substrates were fast red (AP-Red) and diaminobenzidine (DAB) (Roche Ltd) stained. Slides were processed using an automated Roche BenchMark XT staining system according to the manufacturer’s protocol.

## 3 Results

### 3.1 Proportion of immune infiltrating cells and the prognostic efficacy of macrophages

**Figure 1:**
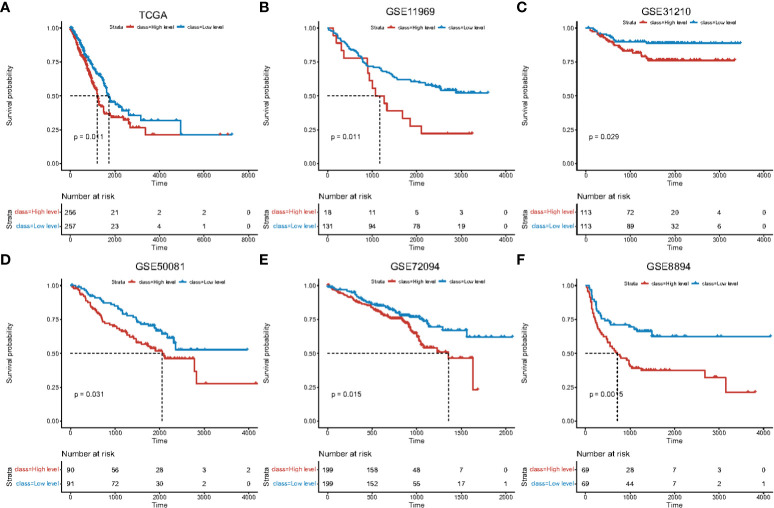
Survival curves of high and low macrophage infiltration in lung adenocarcinoma datasets. Horizontal axis: survival time. Vertical axis: survival probability. Color: level of macrophage infiltration. Survival analysis using **(A)** TCGA data, **(B)** GSE11969, **(C)** GSE31210, **(D)** GSE50081, **(E)** GSE72094, **(F)** GSE8894.

CIBERSORT was used to evaluate the levels of immune-infiltration from different lung adenocarcinoma datasets (TCGA-LUAD, GSE11969, GSE31210, GSE50081, GSE72094, and GSE8894). According to the median macrophage ratio, samples were divided into high- and low-levels of macrophage infiltration. Survival differences between high- and low-groups showed a significant correlation with macrophage infiltration (**Figure 1**; **Supplementary Table 2**_train_cibersort.txt; **Supplementary Table 2**_GSE11969_cibersort.txt; **Supplementary Table 2**_GSE31210_cibersort.txt; **Supplementary Table 2**_GSE50081_cibersort.txt; **Supplementary Table 2**_GSE72094_cibersort.txt; **Supplementary Table 2**_GSE8894_cibersort.txt).

### 3.2 Screening of modules corresponding to macrophages

To identify macrophage-related genes related to infiltration, WGCNA module analysis was performed on the training dataset (**Supplementary Figure 1**, **Supplementary Table 3**_gene_module.txt). Genes corresponding to red modules were named “TAMs-related-genes” for subsequent analysis.

### 3.3 TAMs-related-gene-based clustering analysis, molecular typing and prognostic assessments

We analyzed the expression profiles of TAMs-related-genes in samples from different lung adenocarcinoma datasets (TCGA-LUAD, GSE13213, GSE31210, GSE72094, and GSE8894) to construct consistent clustering profiles. Based on cumulative distribution functions and incremental area maps, we selected stable clusters of TAMs-related-genes to obtain multiple subtypes (**Supplementary Figures 2A–E**, **Supplementary Table 4**_TCGA_consensusClass.csv; **Supplementary Table 4**_GSE13213_consensusClass.csv; **Supplementary Table 4**_GSE31210_consensusClass.csv; **Supplementary Table 4**_GSE72094_consensusClass.csv; **Supplementary Table 4**_GSE8894_consensusClass.csv).

Survival analysis was performed on cluster subtypes from different datasets, revealing significant survival differences (**Supplementary Figures 2F–I**). Dimensionality reduction analysis was performed on each dataset, revealing significant differences in sample characteristics between different subtypes (**Supplementary Figures 2K–O**).

### 3.4 Preprocessing of single-cell data

To further investigate the role of macrophages in lung adenocarcinoma, published single-cell sequencing data of lung adenocarcinoma patients was analyzed (PMC7210975) (32). Gene distribution and mitochondrial gene expression were screened (**Supplementary Figures 3A–C**). Cells with mitochondrial expression ≥20% were identified as dead and removed.

### 3.5 Identification of TAMs in total cells

Markers were used to detect the presence of TAMs in the lung adenocarcinoma single-cell datasets (**Supplementary Figures 3D–I**). TAMs were then extracted and subtype analysis performed to obtain a TAMs subtype map (**Supplementary Figure 3I**).

### 3.6 Screening of differential expression genes among TAMs subsets

To identify marker genes amongst the different TAM subgroups, samples were screened in “Seurat”. Dot and violin plots revealed the top5 marker genes for each TAM subtype (**Supplementary Figure 4**; **Supplementary Table 5**_TAM_marker_genes.txt).

### 3.7 Simulation of dynamic changes in macrophages

“Monocle” was used to identify dynamic changes of macrophages in the tumors and cell polarization (**Supplementary Figures 5A–C**). Cluster 0 could be divided into Cluster 1 and Cluster 2 amongst TAM subtypes. The identified genes were found to regulate differentiation (**Supplementary Figure 5D**). Gene enrichment analysis on the subtypes of TAM showed that Cluster 2 positively correlated with E2F TARGETS and G2M CHECKPOINT, whilst Cluster 4 negatively correlated with these pathways (**Supplementary Figure 5E**).

### 3.8 Screening of prognostic factors based on cell-cluster-markers and TAMS-related-genes using univariate cox regression analysis

**Figure 2:**
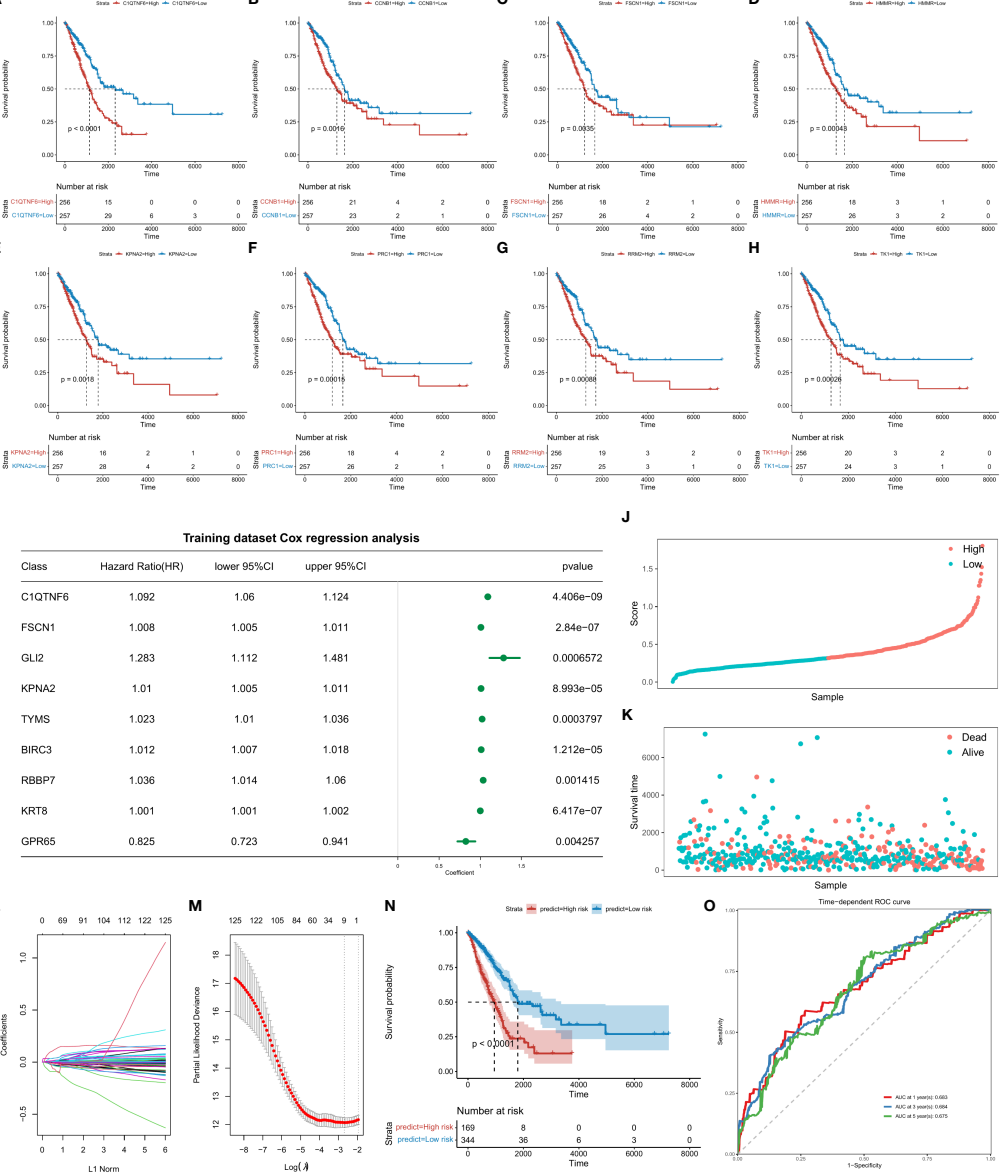
Survival analysis of top8 genes is significantly associated with prognosis. Abscissa axis: survival time. Ordinate axis: survival probability. Colors: differential gene expression. Survival analysis of **(A)** C1QTNF6, **(B)** CCNB1, **(C)**, FSCN1, **(D)** HMMR, **(E)** KPNA2, **(F)** PRC1, **(H)** RRM2, and **(I)** TK1. **(J)** Construction of the risk score model and evaluation of its prognostic efficacy. Forest plots of genes included in the risk score model. Right column: 9 genes included in the risk score model. Left column: corresponding forest plot. **(K)** Risk score plot for cancer samples (line graph). **(L)** Risk score plot for cancer samples (scatter plot graph). **(M)** Dynamic process diagram of variables screened by LASSO regression analysis and selection process diagram of the cross-validation parameter λ. **(N)** Survival analysis of the training dataset. Abscissa axis: survival time; ordinate axis: survival probability. **(O)** ROC curve of training datasets. Abscissa axis: specificity; Ordinate axis: sensitivity. Colors represent different years.

Markers of each TAM subtype and TAMs-related-genes were used to identify genes related to the prognosis. Samples were divided into high- and low-expression groups according to the median of gene expression. Univariate Cox analysis was performed and survival curves of the top8 prognostic genes were displayed (**Figures 2A–H**; **Supplementary Table 6**_cox_significant.txt).

### 3.9 Construction of risk score models and evaluation of the prognostic efficacy

Based on the “GLMNET” of the R package, LASSO (Least Absolute Shrinkage and Selection Operator) regression analysis was used to construct a regression model for the expression matrix of prognosis related genes corresponding to “Cell-Cluster-Markers” and “Tams-Related-Genes”. By analysis, when the value of the freedom degree was 9, the model was accurate (**Figures 2I–O**; **Supplementary Table 7**_forest.univariate_cox.txt, **Supplementary Table 7**_Signature_Coef.txt). The calculation formula of the risk score model are listed as follows:

Risk Score = 0.0354754835*C1QTNF6 (Expression Value) + 0.0023344103* FSCN1 (Expression Value) + 0.0022298189*GLI2 (Expression Value) + 0.0001616254 * KPNA2 (Expression value) + 0.0005176419*TYMS (Expression Value) + 0.0037498174 *BIRC3 (Expression Value) + 0.0033257017*RBBP7 (Expression Value) + 0.0002465129 *KRT8 (Expression Value) - 0.0263442444 *GPR65 (Expression Value). Kaplan-Meier survival curves indicated a significant difference in survival between high and low risk groups. The ROC curve indicated high performance of the risk score model.

### 3.10 Validation of risk score prognostic models in external datasets

**Figure 3:**
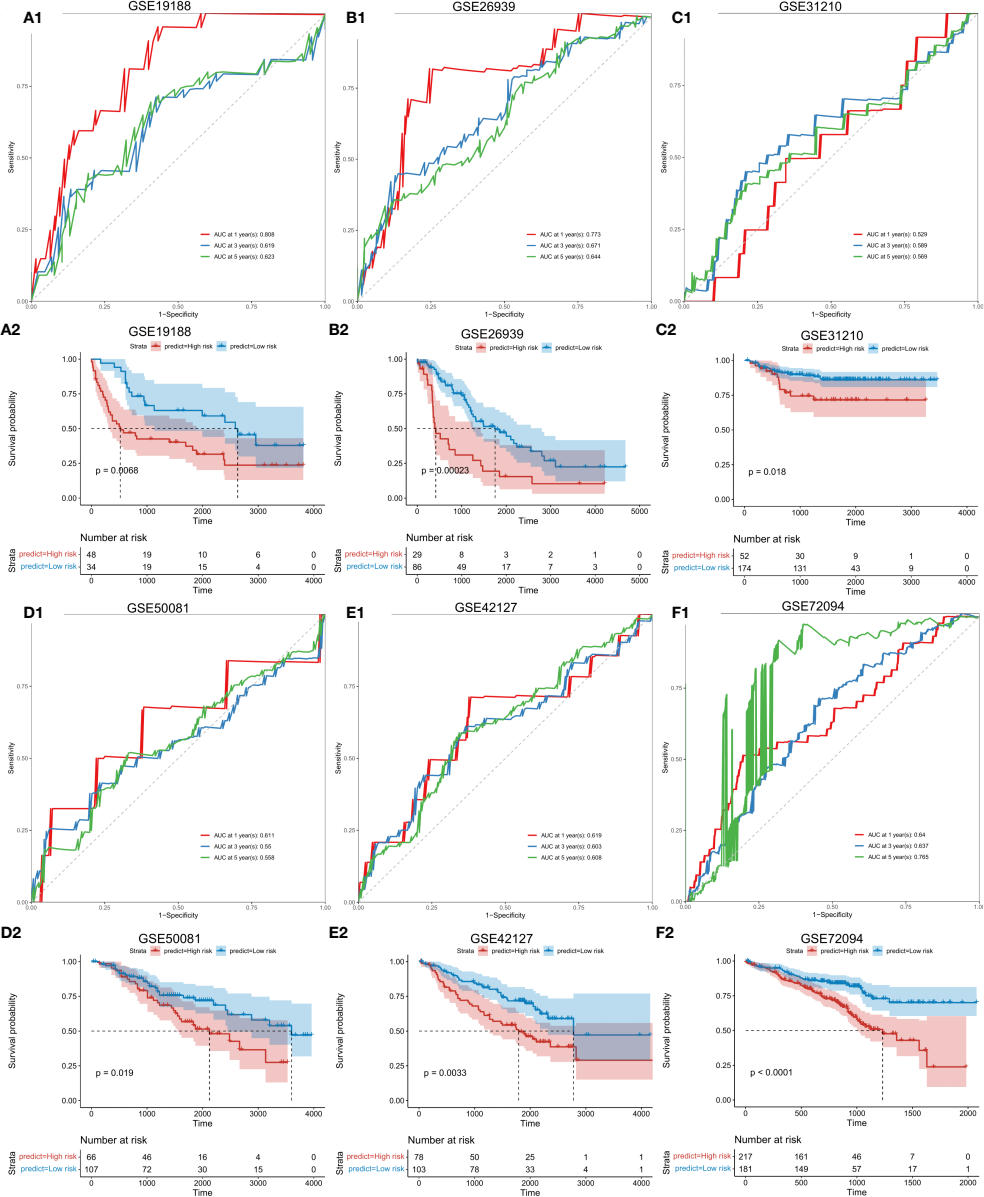
Validation of the risk score model with external independent data. A1: Validation (ROC curve) of the risk score model using external independent data GSE19188; A2: survival analysis. B1: Validation (ROC curve) of the risk score model using GSE26939. B2: Survival analysis using GSE26939. C1: Validation (ROC curve) of the risk score model using GSE31210. C2: Validation (survival analysis) using GSE31210. D1: Validation results (ROC curve) of the risk score model using GSE50081. D2: (survival analysis) of the risk score model using GSE50081. E1: Validation results (ROC curve) of the risk score model using GSE42127. E2: survival analysis using E42127. F1: Validation (ROC curve) of the risk score model using GSE72094. F2: Survival analysis using GSE72094. Abscissa axis: survival time; Ordinate axis: survival probability. Colors: different risk groups.

To further verify the stability of the risk score model, external and independent data GSE19188, GSE26939, GSE31210, GSE50081, GSE42127 and GSE72094 were used to verify predictive efficacy. Through Kaplan-Meier survival analysis, the constructed risk score model performed well for all external data predictions (**Figure 3**).

### 3.11 Robust principal component analysis of risk scoring models in clinical factors

**Figure 4:**
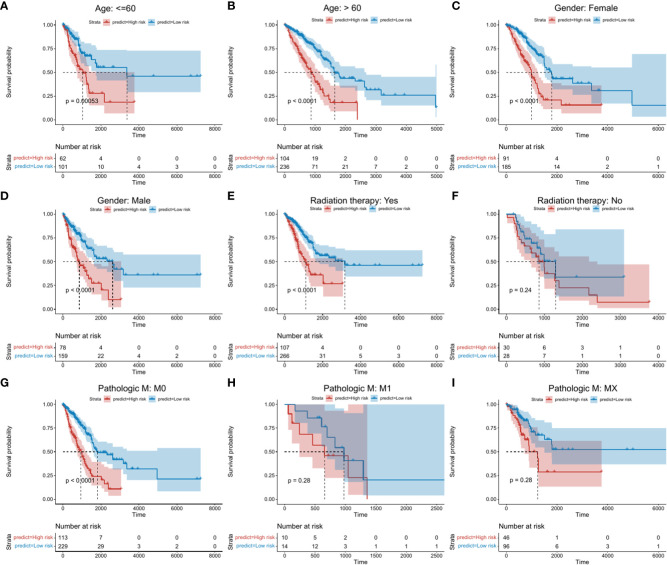
Kaplan-Meier survival analysis between high- and low risk groups. Abscissa axis: survival time; Ordinate axis: survival probability. Colors: different risk groups. **(A)** Kaplan-Meier survival analysis between high- and low-risk groups in those aged ≤ 60 years. **(B)** >60 years. **(C)** Female patients. **(D)** Male patients. **(E)** + Radiation therapy. **(F)** - Radiation therapy. **(G)** M:M0. **(H)** M: M1. **(I)** M: Mx.

To confirm the stability of the risk score model according to clinical characteristics, differences in survival status between high- and low-risk groups in terms of age, gender, radiotherapy, clinical characteristics and Pathlogic M were explored. Significant differences in survival between high- and low-risk groups were observed in those aged ≥ 60 and ≤ 60 years (**Figures 4A, B**; **Supplementary Table 8**_clinical_inf.txt). Similar differences were observed between gender subgroups (**Figures 4C, D**). In the radiotherapy group, differences between high- and low-risk groups were more pronounced (**Figures 4E, F**). In Pathologic M (**Figures 4G, I**), significant differences between high- and low-risk groups were observed for M0, indicative of higher stability.

### 3.12 Differences in risk score models among cancer clinical factors

To investigate the relationship between the risk score model and clinical characteristics, specific features were selected for analysis. The risk score was found to be related to radiation therapy, pathologic T and tumor stage. No significant relationship to age or gender were observed (**Supplementary Figure 6**).

### 3.13 Evaluation of risk score models through univariate and multivariate cox regression analysis

**Figure 5:**
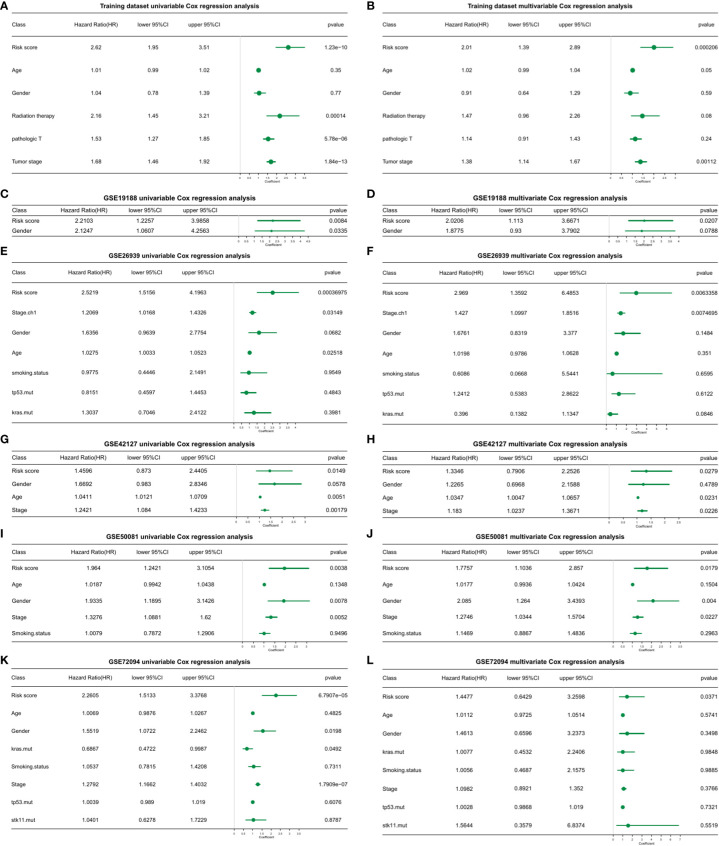
Univariate and multivariate cox regression analysis of the risk score model in training and validation datasets. **(A)** Univariate cox regression analysis. **(B)** Multivariate cox regression analysis. **(C)** GSE19188 univariate cox regression analysis. **(D)** GSE19188 multivariate cox regression analysis. **(E)** GSE26939 univariate cox regression analysis. **(F)** GSE26939 multivariate cox regression analysis. **(G)** GSE42127 univariate cox regression analysis. **(H)** GSE42127 multivariate cox regression analysis. **(I)** GSE50081 univariate cox regression analysis. **(J)** GSE50081 multivariate cox regression analysis. **(K)** GSE72094 univariate cox regression analysis. **(L)** GSE72094 multivariate cox regression analysis.

To determine whether the risk score model could act as an independent prognostic factor for cancer, the “coxph()” function in the R package “survival” was adopted for univariate and multivariate regression analysis on training and test sets, respectively. We found that in all validation and test sets, the p value of the risk score was ≤ 0.05 (**Figure 5**; **Supplementary Table 9**_TCGA_clinical.multivariate_cox.txt; **Supplementary Table 9**_TCGA_ clinical.univariate_cox.txt; **Supplementary Table 9**_GSE19188.multivariate_cox_result.txt; **Supplementary Table 9**_GSE19188.univariate_cox_result.txt; **Supplementary Table 9**_GSE26939. multivariate_cox_result.txt; **Supplementary Table 9**_GSE26939.univariate_cox_result.txt; **Supplementary Table 9**_GSE42127.multivariate_cox_result.txt; **Supplementary Table 9**_GSE42127. univariate_cox_result.txt; **Supplementary Table 9**_GSE50081. multivariate_cox_result.txt; **Supplementary Table 9**_GSE50081.univariate_cox_result.txt; **Supplementary Table 9**_GSE72094. multivariate_cox_result.txt; **Supplementary Table 9**_GSE72094.univariate_cox_result.txt). This indicated that the risk score model was an accurate independent prognostic factor for cancer.

### 3.14 Construction of a nomogram model of risk scores and clinical factors to predict cancer progression

We next sought to apply the risk scoring model to the prediction of cancer progression in the clinic. The R package “rms” was adopted to construct a nomogram using a variety of clinical features. Calibration curves were used to calculate 1, 2, 3, and 5-year survival times (**Supplementary Figure 6**, **Supplementary Table 10**_nomogram_patient_info_part.txt). All survival calibration curves were near the 45° slope, indicating high accuracy of the nomogram.

### 3.15 Prediction of immunotherapy efficacy amongst subtypes

We next investigated whether the risk score model could predict the prognosis of immunotherapy. Data were calculated using the risk score model and the K-M survival status between high- and low-risk groups evaluated (**Supplementary Figure 7A**, **Supplementary Table 11**_Immune_treatment.xlsx). Upon statistical analysis of the distribution of CR/PR and PD/SD, the proportion of treatment response rates significantly differed between high- and low-risk groups (**Supplementary Figure 7B**, chi-square test p= 0.004133). No significant differences in the risk scores between the different treatment response groups were observed (**Supplementary Figure 7C**).

### 3.16 Overall survival analyses of M1 and M2 macrophage subtypes in patients with lung cancer

A total of 32 patients with lung cancer were evaluated for M1 and M2 macrophage subtypes. Samples were stained using double-labeled IHC. The majority of patients were in pathological Stage II (62.5%) and the dominant histopathological type was adenocarcinoma (68.8%). The clinicopathological characteristics of the lung cancer patients are shown in (**Table 1**).

**Table 1:** Basic characteristics of enrolled clinical samples.

Characteristic	levels	Overall
n		32
Age, n (%)	>65	12 (37.5%)
	≤65	20 (62.5%)
Gender, n (%)	Female	16 (50%)
	Male	16 (50%)
T stage, n (%)	T1	5 (15.6%)
	T2	20 (62.5%)
	T3	7 (21.9%)
N stage, n (%)	N0	21 (65.6%)
	N1	8 (25%)
	N2	3 (9.4%)
Pathological Stage, n (%)	I	8 (25%)
	II	20 (62.5%)
	III	4 (12.5%)
Histologic type, n (%)	Adenomcarcinoma	22 (68.8%)
	Mucoepidermoid carcinoma	2 (6.2%)
	Squamous cell carcinoma	8 (25%)

**Figure 6:**
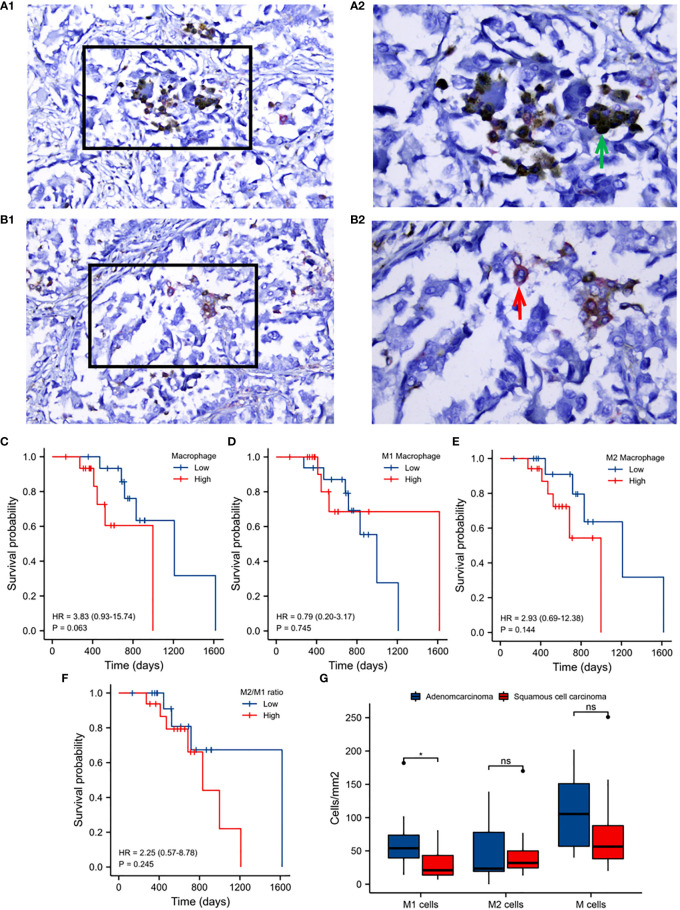
Validation analysis of clinical samples. A1: Immunohistochemical staining of M1 macrophages. A2: Enlargement of the boxed regions. B1: Immunohistochemical staining of M2 macrophages. B2: Enlargement of the boxed regions. Images were obtained at 40×10 magnification under a light microscope. **(C)** Kaplan-Meier survival analysis of macrophage infiltration. **(D)** M1 macrophage infiltration. **(E)** M2 macrophage infiltration. **(F)** M2 to M1 macrophage infiltration. **(G)** M1, M2 and total macrophage infiltration between adenocarcinoma and squamous cell carcinoma. Horizontal axis: survival time. Vertical axis: survival probability. Colors: macrophage infiltration.

To identify M1 and M2 macrophage subtypes, CD68 and CD163 antibodies were used for double-labeled IHC staining. CD68 (brown/yellow) as a surface marker for all macrophages primarily localized to the cytoplasm, whilst CD163 (red) localized to the plasma membrane. M2 macrophages were identified through double staining for CD68 and CD163. M1 macrophages were identified through staining with CD68 alone. Representative IHC images are shown in (**Figures 6A, B**).

The prognostic value of macrophage infiltration was next evaluated. Total macrophages, M2 to M1, and M2 macrophage infiltration were identified as detrimental to patient survival (**Figures 6C, E, F**), whilst M1 macrophage infiltration was beneficial to prognosis (**Figure 6D**). The infiltration of M1 macrophages in adenocarcinoma was significantly higher than that in squamous cell carcinoma of lung cancer. No significant differences in M2 nor total macrophage infiltration were observed between these two histological subtypes (**Figure 6G**).

## 4 Discussion

Macrophages with different phenotypes are frequently cited as indicators of the prognosis of lung cancer patients and the efficacy of immunotherapy (17–24). In our preliminary analyses, macrophage infiltration, rarely reported in lung cancer, had a significant detrimental effect on the prognosis of lung cancer patients (**Figure 1**). These data were consistent across cohorts (**Figures 1A–F**) and further verified in follow-up immunohistochemical analysis of clinical samples (**Figure 6C**). Collectively, these data highlight how macrophages not only act as innate immune cells to regulate immunological responses (23), but play an important role in the prognosis of lung cancer. This lays the foundation for subsequent module analysis based on macrophage infiltration (**Supplementary Figure 1**).

Based on the expression profiles of TAMs-related-genes, a consistent clustering profile was constructed (**Supplementary Figures 2A–E**). Significant differences in both survival analysis and PCA (**Supplementary Figures 2F–O**) were observed. These apparent differences were further identified in single-cell data (**Supplementary Figure 3**) confirming the importance of macrophages to the prognosis of lung cancer patients (17–24, 32). These data also highlight the need for further refinement of relevant factors to more favorably evaluate patient prognosis.

Given the advantages and progress of single-cell sequencing in lung cancer immunity (33–35), the single-cell data was further analyzed (**Supplementary Figures 4**, **5**, and **Figure 2**) (32). Based on cell-cluster-markers and TAMs-related-genes, TOP 8 genes (C1QTNF6, CCNB1, FSCN1, HMMR, KPNA2, PRC1, RRM2, and TK1) significantly associated with prognosis were obtained (**Figure 2**). These have obvious benefits to clinicians for the assessment of patient prognosis (36–49). The same data were used to construct a risk score model containing 9 factors (C1QTNF6, FSCN1, KPNA2, GLI2, TYMS, BIRC3, RBBP7, KRT8, and GPR65) for prognostic evaluation (**Figure 2**) (50–55). The model was validated using external data cohorts (**Figure 3**) and identified as robust and accurate for prognostic evaluation (**Figure 4**). Significant differences in the risk scores were observed for clinical characteristics including radiation therapy, pathologic T, and Tumor stage (**Supplementary Figures 6A–E**). This further highlighted the efficiency of the risk score to predict therapeutic efficacy.

Through univariate and multivariate cox regression analysis, the risk score model held utility as an independent prognostic factor for cancer, further affirming its clinical benefits (**Figure 5**). Furthermore, cancer progression could be more accurately predicted using nomogram models constructed based on risk scores and clinical factors (**Supplementary Figures 6F–J**). For prognostic assessments of immunotherapy, the risk score model could also act as an accurate evaluation tool (**Supplementary Figure 7**). Upon immunohistochemical analysis of clinical tissue samples to verify the correlation between the macrophage phenotype and patient prognosis, similar conclusions were obtained (**Table 1**; **Figures 1**, **6**). Macrophage infiltration, particularly for the M2 phenotype, were not conducive to the prognosis and survival of patients, consistent with previous studies (20–24, 56, 57).

We used WGCNA to identify macrophage infiltration-related module genes and single-cell sequencing of lung adenocarcinoma tissue to identify marker genes of macrophage subtypes. This permitted the construction of a risk assessment model with high prognostic efficacy. The model performed well on external and independent datasets. Immunohistochemistry analysis of clinical samples were consistent with our data. We therefore infer that the risk score has both high clinical practicability and application.

## 5 Conclusion

Macrophage infiltration was negatively correlated with prognosis for patients with lung adenocarcinoma. Based on cell-cluster-markers and TAMs-related-genes, both TOP8 genes (C1QTNF6, CCNB1, FSCN1, HMMR, KPNA2, PRC1, RRM2, TK1) and the risk score model containing 9 risk factors (C1QTNF6, FSCN1, KPNA2, GLI2, TYMS, BIRC3, RBBP7, KRT8, GPR65) had a high efficacy for the prediction of prognosis.

## Data availability statement

The datasets presented in this study can be found in online repositories. The names of the repository/repositories and accession number(s) can be found in the article/**Supplementary Material**.

## Ethics statement

The ethics involved in the clinical samples in this study have been approved by the Ethics Committee of Qianfoshan Hospital of Shandong Province (2022-S527). The patients/participants provided their written informed consent to participate in this study.

## Author contributions

Corresponding authors (YT) conceived the idea and designed this study. TY was responsible for the final submission. YT, CZ and HZ were responsible for data collection, partial data analysis and drafting the manuscript. HL performed immunohistochemical staining of the clinical tissue and related data analysis. FK, SK, and FC were reviewed the manuscript and were responsible for data corrections. All authors contributed to the article and approved the submitted version.

## Funding

The study was funded by Clinical Research Fund of Shandong Medical Association Qilu Special Project (YXH2022ZX02016; YT), and Jinan Clinical Medicine Technology Innovation Program (YT).

## Conflict of interest

The authors declare that the research was conducted in the absence of any commercial or financial relationships that could be construed as a potential conflict of interest.

## Publisher’s note

All claims expressed in this article are solely those of the authors and do not necessarily represent those of their affiliated organizations, or those of the publisher, the editors and the reviewers. Any product that may be evaluated in this article, or claim that may be made by its manufacturer, is not guaranteed or endorsed by the publisher.

## Supplementary material

Supplementary Figure 1 Screening of modules corresponding to macrophages using WGCNA (Weighted Gene Co-Expression Network Analysis). **(A)** The cluster dendrogram of modular genes associated with macrophage infiltration. **(B)** Heatmap of module-trait relationships associated with macrophage infiltration. **(C)** Module genes relevant heatmap related to macrophage infiltration. **(D)** Scatter plot of correlation between modules and gene features.

Supplementary Figure 2 Sample clustering, molecular typing, and prognosis evaluation analyses based on TAMs-related-genes. **(A)** Subtypes of clustering profiles obtained through analysis of the expression profiles of TAMs-related-genes in TCGA-LUAD samples (consensus matrix k=3). **(B)** Subtypes of consistent clustering profiles obtained through analysis of the expression profiles of TAMs-related-genes in GSE13213 samples (consensus matrix k=3). **(C)** Subtypes of the consistent clustering profiles obtained from GSE31210 samples (consensus matrix k=2). **(D)** Clustering profiles from GSE72094 samples (consensus matrix k=2). **(E)** GSE8894 samples (consensus matrix k=3). **(F)** Survival analysis of different cluster subtypes in TCGA-LUAD samples. **(G)** Results of survival analysis of different cluster subtypes in GSE13213 samples; The horizontal axis represents survival time; the vertical axis represents survival probability; Curves with different colors represent different cluster subtypes. **(H)** Results of survival analysis of different cluster subtypes in GSE31210 samples; The horizontal axis represents survival time; the vertical axis represents survival probability; Curves with different colors represent different cluster subtypes. **(I)** Results of survival analysis of different cluster subtypes in GSE72094 samples; The horizontal axis represents survival time; the vertical axis represents survival probability; Curves with different colors represent different cluster subtypes. **(J)** Results of survival analysis of different cluster subtypes in GSE8894 samples; The horizontal axis represents survival time; the vertical axis represents survival probability; Curves with different colors represent different cluster subtypes. **(K)** Results of principal component analysis (PCA) on TCGA-LUAD samples; **(L)** Results of principal component analysis (PCA) on GSE13213 samples; **(M)** Results of principal component analysis (PCA) on GSE31210 samples; **(N)** Results of principal component analysis (PCA) on GSE72094 samples; **(O)** Results of principal component analysis (PCA) on GSE8894 samples.

Supplementary Figure 3 Single-cell data. **(A)** Number of genes expressed in cells; **(B)** Total counts. **(C)** Mitochondrial gene expression. **(D–I)** UMAP (Uniform Manifold Approximation and Projection) dimensionality reduction analysis results of TAM subgroups. **(D)** C1QA. **(E)** C1QB. **(F)** APOE. **(G)** C1QC. **(H)** Analysis of TAM clusters. **(I)** Subtypes derived from re-clustering of TAM cell populations.

Supplementary Figure 4 Screening of differentially expressed genes amongst tumor macrophage subsets. **(A)** Dotplot of Top5 maker genes of each subtype. Abscissa axis: marker genes. Ordinate axis: top5 of TAM subtypes. Colors: mean expression per-group; Dot sizes represent the fraction of cells in each group (%). **(B–G)** Violin plots of the expression of the top5 marker genes. Abscissa axis: different TAM subtypes. Ordinate axis: gene expression.

Supplementary Figure 5 Pseudo-chronological analysis of tumor macrophages for simulation of the dynamic changes of macrophages. **(A–C)** Differential states according to monocle trajectory analysis, distribution of TAMs in trajectories, and pseudo-sequences of differentiation. **(D)** Genes influencing differentiation states in the clusters. Left column: different clusters. Right column: names of genes. **(E)** Pathway enrichment analysis of different TAM subtypes. Color: correlation; Red: positive correlation; Blue: negative correlation. Numerical values: correlation p-value.

Supplementary Figure 6 Comparison of risk scores corresponding to the clinical characteristics of the different groups. **(A)** Age, **(B)** Gender, **(C)** Radiation therapy. **(E)** Pathologic T cells; **(E)** Tumor stage. Abscissa axis: Different groups. Ordinate axis: risk scores. **(F)** Nomogram model for risk scores and clinical factors according to the clinical characteristics of prognosis. **(G)** Calibration curve for 1-year survival. **(H)**. 2-year survival. (I) 3-year survival. **(J)** 5-year survival. Abscissa axis: predicted probability of survival. Ordinate axis: actual survival.

Supplementary Figure 7 Assessment of immunotherapy prognosis according to the risk score. **(A)** Survival analysis of immunotherapy responses in the training set. Abscissa axis: survival time. Ordinate axis: survival probability. Colors represent different risk groups. **(B)** Comparative analysis of the proportion of treatment response states between high and low risk groups. **(C)** Comparative analysis of risk scores for different treatment response states.

## Abbreviations

UMAP, Uniform Manifold Approximation and Projection; NSCLC, non-small cell lung cancer; SCLC, small cell lung cancer; LUAD, lung adenocarcinoma; WGCNA, Weighted Gene Co-Expression Network Analysis; TAM, tumor-associated macrophage; GSVA, Gene Set Variation Analysis; FFPE, formalin fixation and paraffin embedding; IHC, immunohistochemical; DAB, diaminobenzidine; DEGs, Differential Expression Genes; LASSO, Least Absolute Shrinkage and Selection Operator.
