# Supplementary figures and images for: Significance of macrophage infiltration in the prognosis of lung adenocarcinoma patients evaluated by scRNA and bulkRNA analysis
Source: Front Immunol. 2022 Oct 12;13:1028440. doi: 10.3389/fimmu.2022.1028440 (PMC9597471; doi:10.3389/fimmu.2022.1028440)

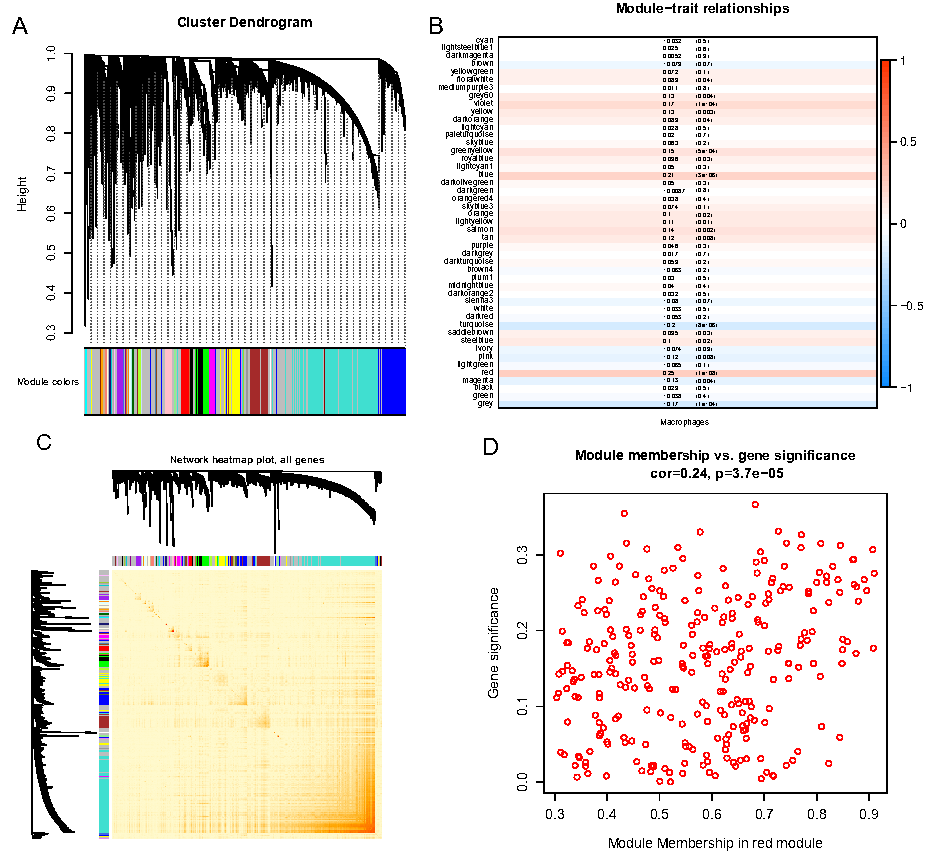

Supplement: Supplementary Figure 1 — Screening of modules corresponding to macrophages using WGCNA (Weighted Gene Co-Expression Network Analysis). (A) The cluster dendrogram of modular genes associated with macrophage infiltration. (B) Heatmap of module-trait relationships associated with macrophage infiltration. (C) Module genes relevant heatmap related to macrophage infiltration. (D) Scatter plot of correlation between modules and gene features. [file Image_1.tiff]

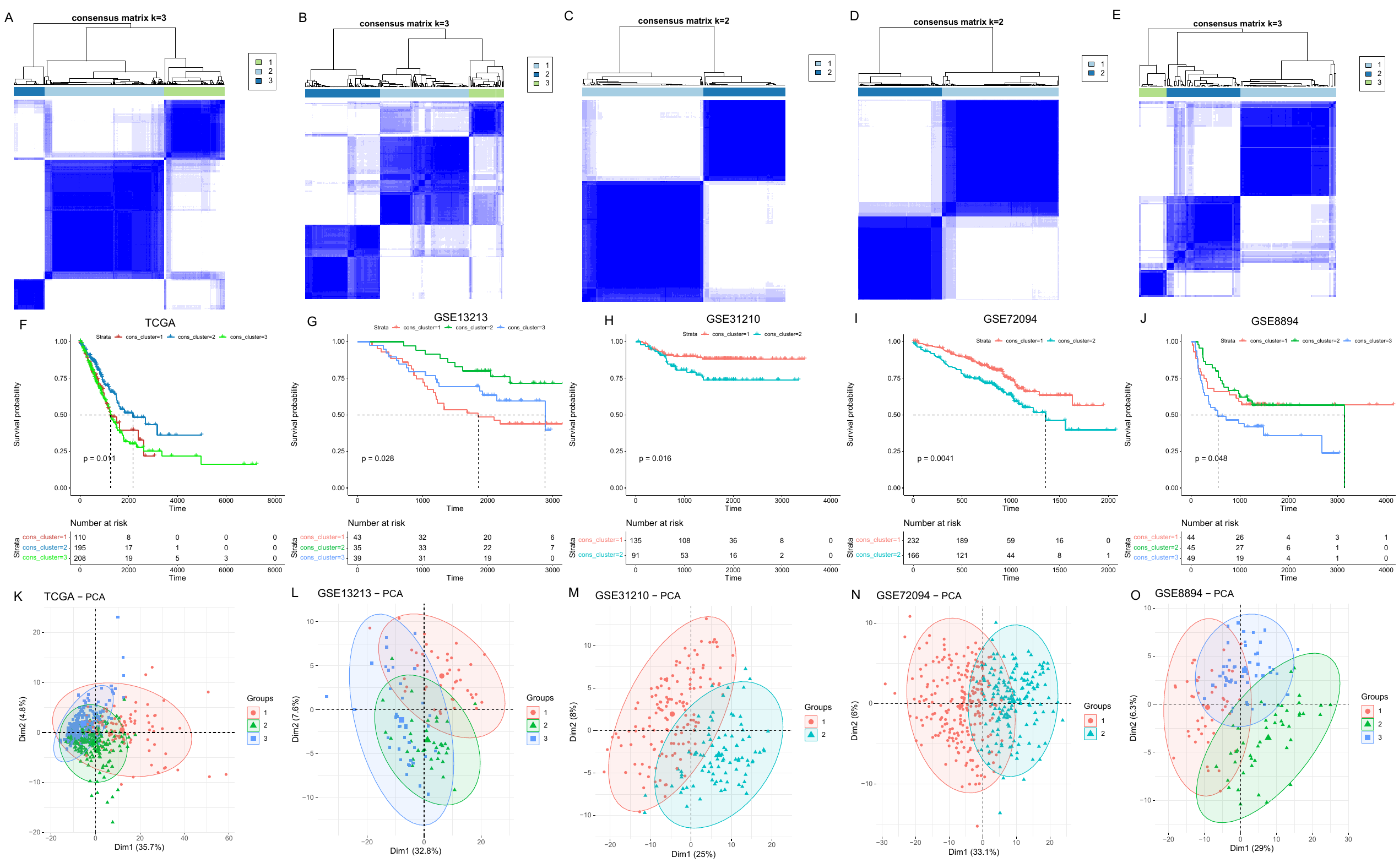

Supplement: Supplementary Figure 2 — Sample clustering, molecular typing, and prognosis evaluation analyses based on TAMs-related-genes. (A) Subtypes of clustering profiles obtained through analysis of the expression profiles of TAMs-related-genes in TCGA-LUAD samples (consensus matrix k=3). (B) Subtypes of consistent clustering profiles obtained through analysis of the expression profiles of TAMs-related-genes in GSE13213 samples (consensus matrix k=3). (C) Subtypes of the consistent clustering profiles obtained from GSE31210 samples (consensus matrix k=2). (D) Clustering profiles from GSE72094 samples (consensus matrix k=2). (E) GSE8894 samples (consensus matrix k=3). (F) Survival analysis of different cluster subtypes in TCGA-LUAD samples. (G) Results of survival analysis of different cluster subtypes in GSE13213 samples; The horizontal axis represents survival time; the vertical axis represents survival probability; Curves with different colors represent different cluster subtypes. (H) Results of survival analysis of different cluster subtypes in GSE31210 samples; The horizontal axis represents survival time; the vertical axis represents survival probability; Curves with different colors represent different cluster subtypes. (I) Results of survival analysis of different cluster subtypes in GSE72094 samples; The horizontal axis represents survival time; the vertical axis represents survival probability; Curves with different colors represent different cluster subtypes. (J) Results of survival analysis of different cluster subtypes in GSE8894 samples; The horizontal axis represents survival time; the vertical axis represents survival probability; Curves with different colors represent different cluster subtypes. (K) Results of principal component analysis (PCA) on TCGA-LUAD samples; (L) Results of principal component analysis (PCA) on GSE13213 samples; (M) Results of principal component analysis (PCA) on GSE31210 samples; (N) Results of principal component analysis (PCA) on GSE72094 samples; (O) Resu [file Image_2.tiff]

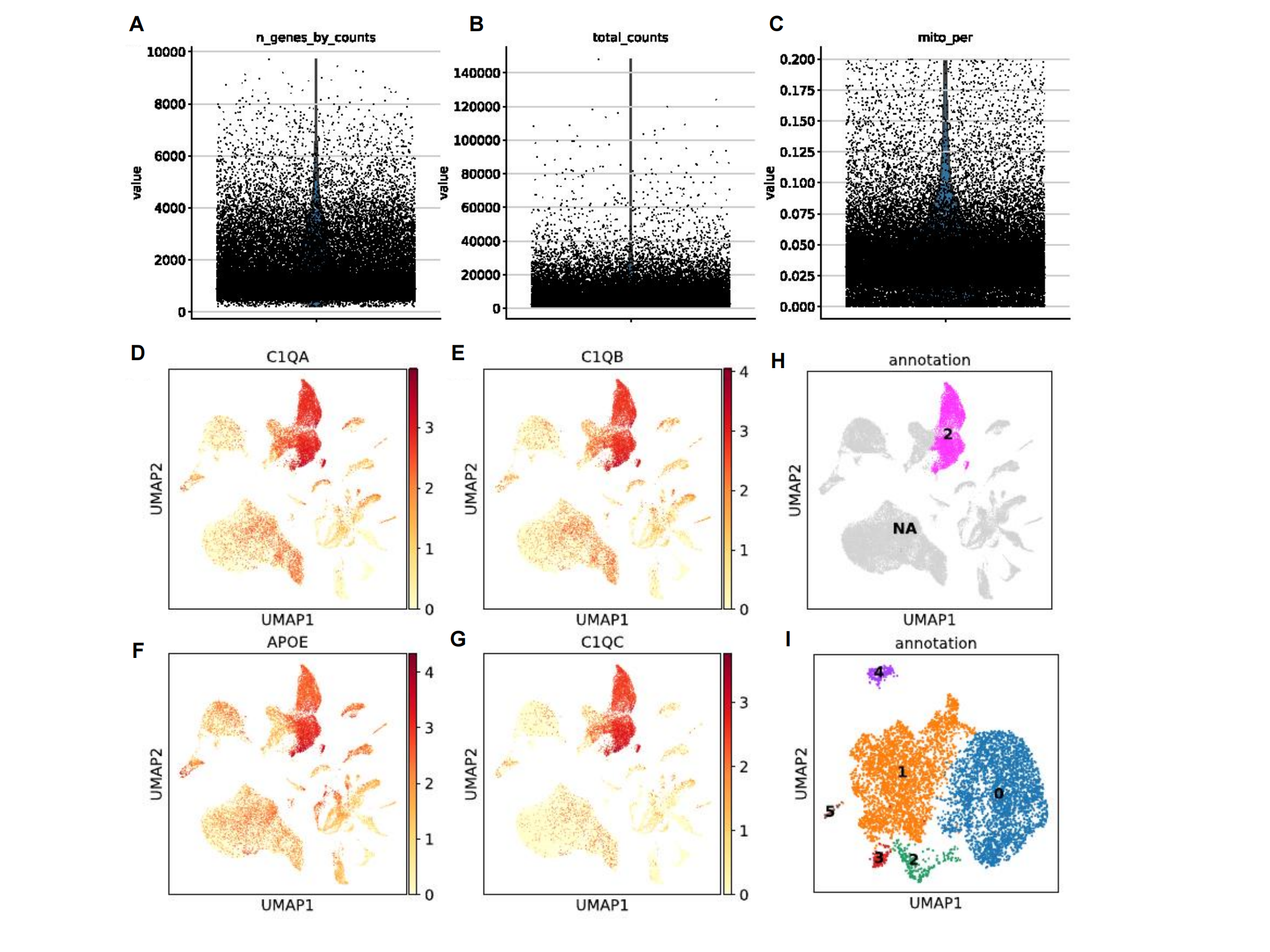

Supplement: Supplementary Figure 3 — Single-cell data. (A) Number of genes expressed in cells; (B) Total counts. (C) Mitochondrial gene expression. (D–I) UMAP (Uniform Manifold Approximation and Projection) dimensionality reduction analysis results of TAM subgroups. (D) C1QA. (E) C1QB. (F) APOE. (G) C1QC. (H) Analysis of TAM clusters. (I) Subtypes derived from re-clustering of TAM cell populations. [file Image_3.tif]

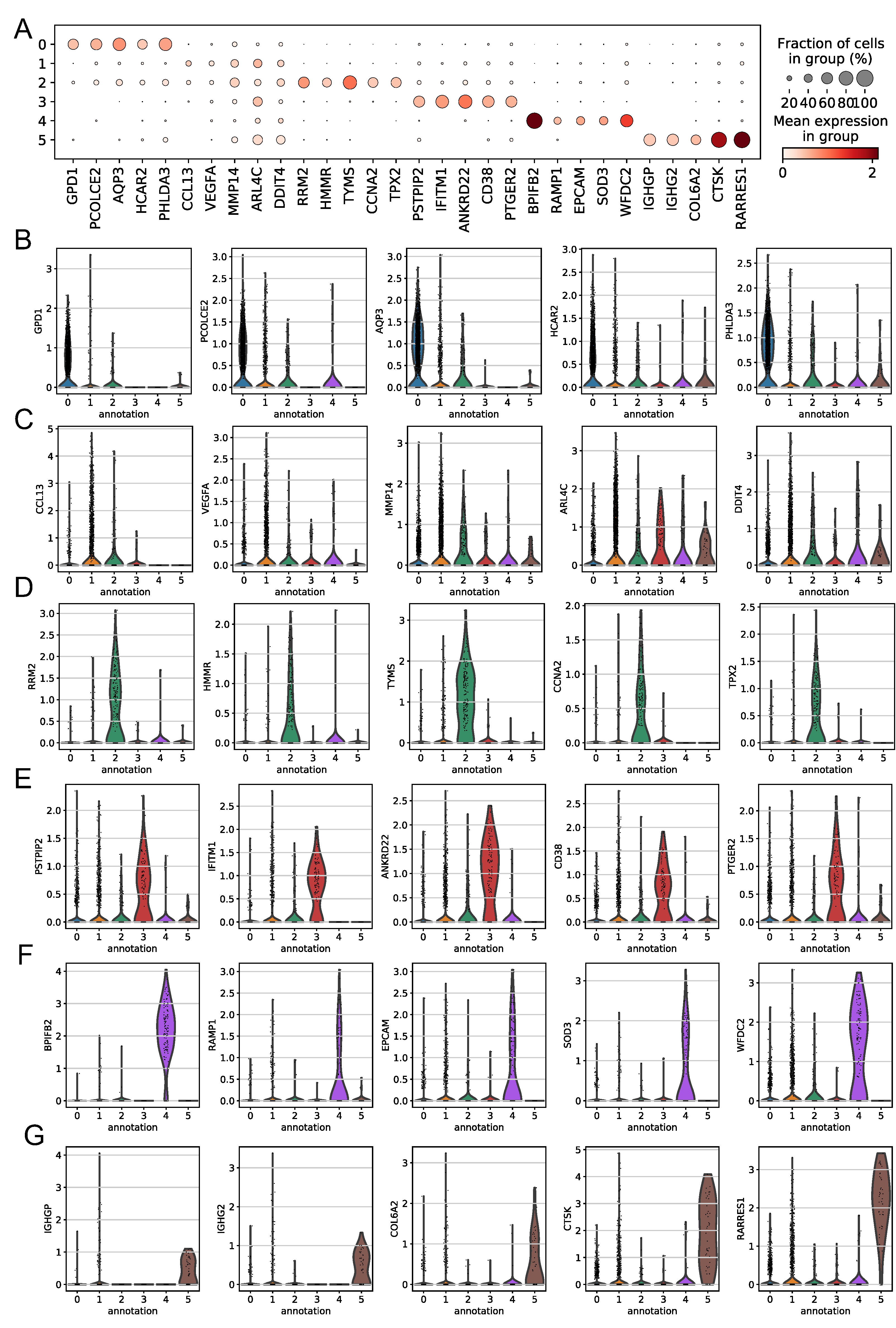

Supplement: Supplementary Figure 4 — Screening of differentially expressed genes amongst tumor macrophage subsets. (A) Dotplot of Top5 maker genes of each subtype. Abscissa axis: marker genes. Ordinate axis: top5 of TAM subtypes. Colors: mean expression per-group; Dot sizes represent the fraction of cells in each group (%). (B–G) Violin plots of the expression of the top5 marker genes. Abscissa axis: different TAM subtypes. Ordinate axis: gene expression. [file Image_4.tiff]

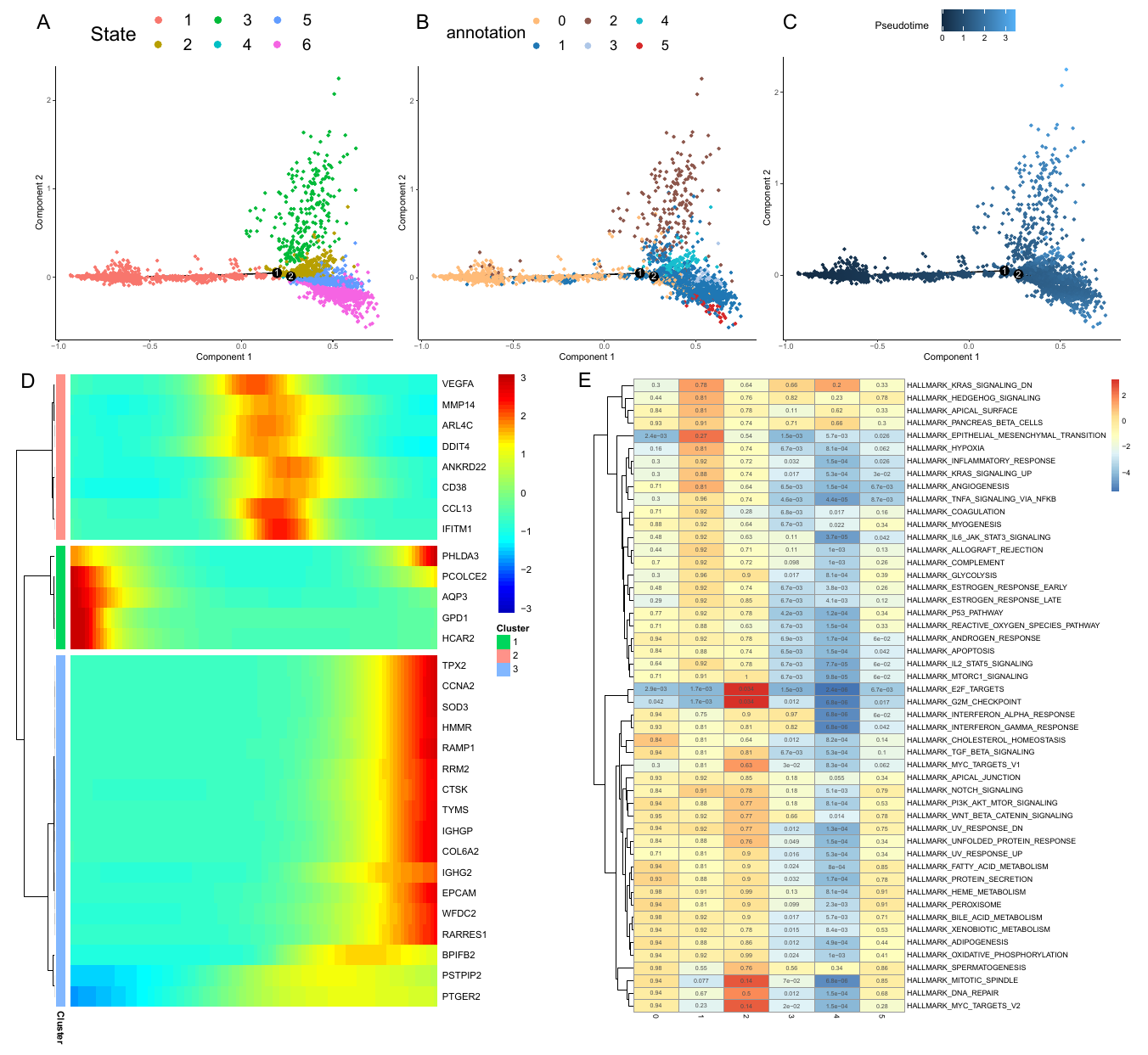

Supplement: Supplementary Figure 5 — Pseudo-chronological analysis of tumor macrophages for simulation of the dynamic changes of macrophages. (A–C) Differential states according to monocle trajectory analysis, distribution of TAMs in trajectories, and pseudo-sequences of differentiation. (D) Genes influencing differentiation states in the clusters. Left column: different clusters. Right column: names of genes. (E) Pathway enrichment analysis of different TAM subtypes. Color: correlation; Red: positive correlation; Blue: negative correlation. Numerical values: correlation p-value. [file Image_5.tiff]

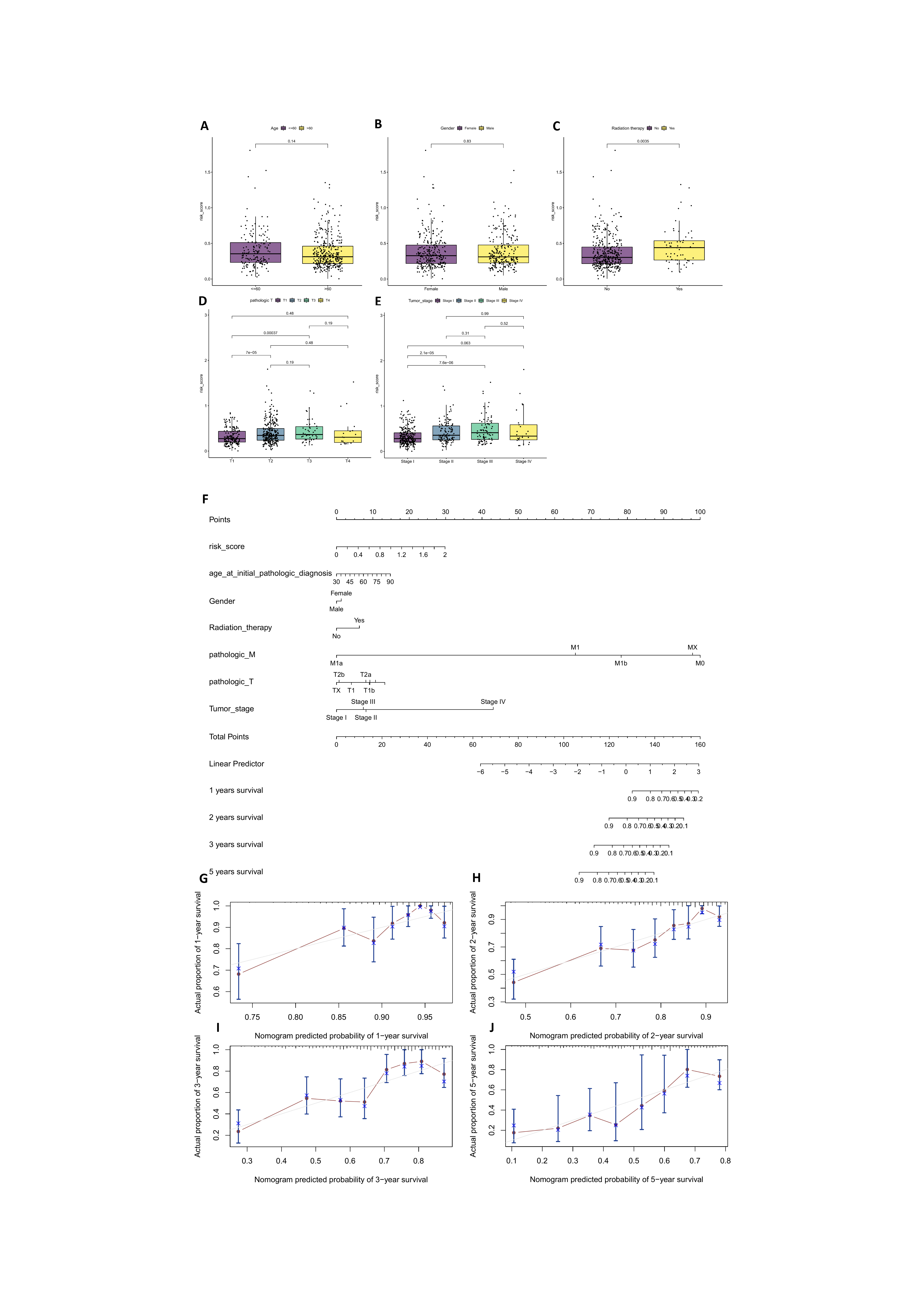

Supplement: Supplementary Figure 6 — Comparison of risk scores corresponding to the clinical characteristics of the different groups. (A) Age, (B) Gender, (C) Radiation therapy. (E) Pathologic T cells; (E) Tumor stage. Abscissa axis: Different groups. Ordinate axis: risk scores. (F) Nomogram model for risk scores and clinical factors according to the clinical characteristics of prognosis. (G) Calibration curve for 1-year survival. (H). 2-year survival. (I) 3-year survival. (J) 5-year survival. Abscissa axis: predicted probability of survival. Ordinate axis: actual survival. [file Image_6.tif]

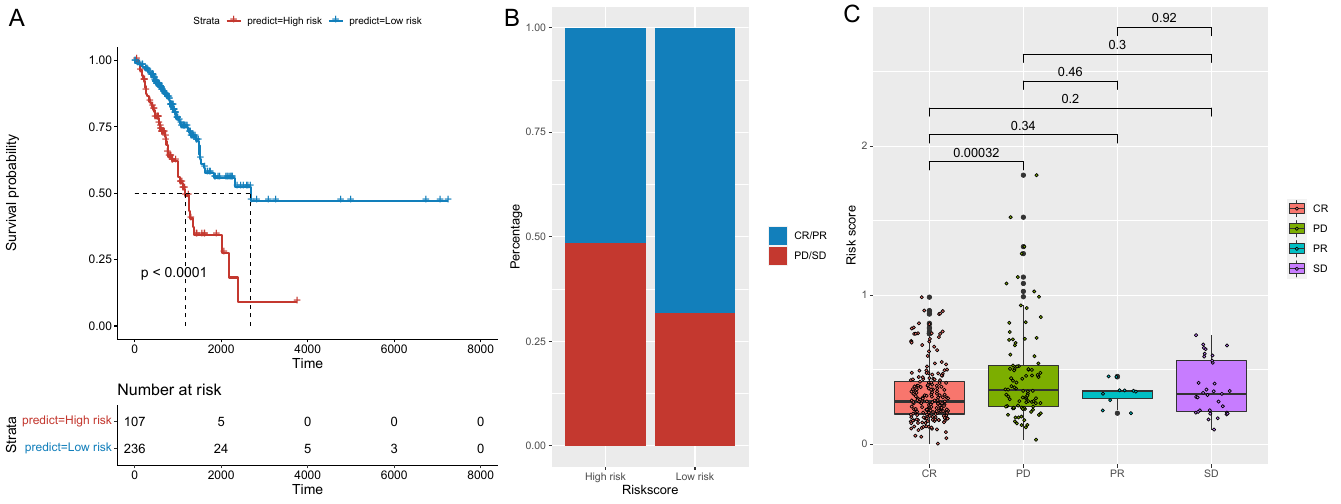

Supplement: Supplementary Figure 7 — Assessment of immunotherapy prognosis according to the risk score. (A) Survival analysis of immunotherapy responses in the training set. Abscissa axis: survival time. Ordinate axis: survival probability. Colors represent different risk groups. (B) Comparative analysis of the proportion of treatment response states between high and low risk groups. (C) Comparative analysis of risk scores for different treatment response states. [file Image_7.tiff]
